# Hypersensitivity reactions to contrast media: Part 1. Management of immediate and non-immediate hypersensitivity reactions in adults. Updated guidelines by the ESUR Contrast Media Safety Committee

**DOI:** 10.1007/s00330-025-11675-1

**Published:** 2025-05-27

**Authors:** Aart J. van der Molen, Annick A. J. M. van de Ven, Francisco Vega, Ilona A. Dekkers, Remy W. F. Geenen, Marie-France Bellin, Michele Bertolotto, Torkel B. Brismar, Olivier Clément, Jean-Michel Correas, Katerina Deike, Gertraud Heinz, Andreas H. Mahnken, Carlo A. Mallio, Carlo C. Quattrocchi, Alexander Radbruch, Peter Reimer, Giles Roditi, Laura Romanini, Carmen Sebastià, Fulvio Stacul

**Affiliations:** 1 Department of Radiology, Leiden University Medical Center, Leiden, The Netherlands; 2 Department of Internal Medicine, Division of Allergology, University Medical Center Groningen, Groningen, The Netherlands; 3 Department of Allergy, Hospital Universitario de la Princesa, Madrid, Spain; 4 Department of Radiology, Amsterdam University Medical Center, Amsterdam, The Netherlands; 5 Department of Radiology, Northwest Clinics, Alkmaar, The Netherlands; 6 University Paris Saclay, AP-HP, University Hospital Bicêtre, Department of Radiology, BioMaps, Le Kremlin-Bicêtre, France; 7 Department of Radiology, Cattinara Hospital, University of Trieste, Trieste, Italy; 8 Department of Clinical Science, Intervention and Technology, Unit of Radiology, Karolinska Institute and Department of Radiology, Karolinska University Hospital in Huddinge, Stockholm, Sweden; 9 Université de Paris, AP-HP, Hôpital Européen Georges Pompidou, DMU Imagina, Service de Radiologie, Paris, France; 10 Université de Paris, AP-HP, Groupe Hospitalier Necker, DMU Imagina, Service de Radiologie, Paris, France; 11 Clinic for Diagnostic and Interventional Neuroradiology, University Clinic Bonn, and German Center for Neurodegenerative Diseases, DZNE, Bonn, Germany; 12 Athinoula A. Martinos Center for Biomedical Imaging, Massachusetts General Hospital, Charlestown, MA USA; 13 Department of Radiology, Landesklinikum St Pölten, St Pölten, Austria; 14 Department of Diagnostic and Interventional Radiology, Marburg University Hospital, Marburg, Germany; 15 Fondazione Policlinico Universitario Campus Bio-Medico, Roma, Italy; 16 Centre for Medical Sciences CISMed, University of Trento, Trento, Italy; 17 Department of Radiology, Institute for Diagnostic and Interventional Radiology, Klinikum Karlsruhe, Karlsruhe, Germany; 18 Department of Radiology, Glasgow Royal Infirmary, Glasgow, UK; 19 Department of Radiology, ASST Cremona, Cremona, Italy; 20 Department of Radiology, Hospital Clinic de Barcelona, Barcelona, Spain; 21 Department of Radiology, Ospedale Maggiore, Trieste, Italy

**Keywords:** Contrast media, Hypersensitivity (immediate), Hypersensitivity (delayed), Clinical protocols, Practice guideline

## Abstract

**Abstract:**

Hypersensitivity reactions to iodine-based, gadolinium-based and ultrasound contrast agents are a heterogeneous group that occur infrequently and can be classified as either immediate or non-immediate. In imaging, most are mild or moderate, while severe reactions are very rare. A standardized risk assessment and close patient monitoring are crucial. In addition to liberal use of supportive treatment by oxygen and volume expansion, the main drugs for treatment of individual reactions include intramuscular adrenaline and H1-antihistamines, with limited indications for short-acting β2-agonists (SABA) like salbutamol or albuterol. As these reactions occur infrequently, these situations often create stress and confusion. During treatment, the stability or progression of the reaction should be constantly assessed, assigning a specific task to each team member. In addition, detailed documentation and correct classification of the severity of such reactions will help improve the risk management in future imaging studies. Regular hands-on training for all personnel involved in managing these reactions is mandatory. To help, the ESUR Contrast Media Safety Committee has reviewed the literature on hypersensitivity reactions and updated its guidance on how to appropriately manage, treat, and document immediate and non-immediate hypersensitivity reactions to contrast media in adults.

**Key Points:**

***Question*** *Hypersensitivity reactions to contrast media may require immediate action to prevent further damage to the patient. Guidance on management would help improving preparedness.*

***Findings*** *Clear treatment protocols with cards or flowcharts are mandatory. Intramuscular adrenaline is the most important drug for treatment. Documentation should be agent-specific and standardized.*

***Clinical relevance*** *The ESUR Contrast Media Safety Committee has updated its guidance on how to appropriately manage, treat, and document immediate and non-immediate hypersensitivity reactions to contrast media in adults.*

## Introduction

The Contrast Media Safety Committee (CMSC) of the European Society of Urogenital Radiology (ESUR) decided to update its practice guideline on the prevention of hypersensitivity reactions (HR) to contrast media (CM).

Immediate (acute) hypersensitivity reactions (IHR) to contrast media are infrequent, but they often create stress and confusion. In Radiology, most of them are mild to moderate, while severe reactions are very rare. A standardized risk assessment is mandatory prior to the examination. Treatment of a (potentially serious) HR may vary depending on the location, available personnel expertise, and the availability of equipment and drugs. New monoclonal antibody treatments targeting specific allergological pathways have not yet been established for treating CM reactions.

Non-immediate (delayed) hypersensitivity reactions (NIHR) are heterogeneous. Because of the self-limiting character of most cutaneous NIHR to CM, the traditional mainstay of treatment follows that of cutaneous NIHR to other drugs: withdrawal of the drug combined with symptomatic treatment.

The purpose of Part 1 of this guideline is to outline how to appropriately manage, treat and document IHR and NIHR to contrast media, and to promote standardized treatment availability and preparedness.

## Materials and methods

Two systematic literature analyses were performed using PubMed and Embase databases from January 1985 until May 2022. Search criteria for immediate HR included (synonyms of) “contrast media,” “immediate,” “hypersensitivity,” “anaphylaxis,” “treatment,” and “management.” Search criteria for non-immediate HR included (synonyms of) “contrast media,” “non-immediate,” “hypersensitivity,” “treatment,” “management,” “severe cutaneous adverse reaction,” “acute generalized exanthematous pustulosis,” “drug reaction with eosinophilia and systemic symptoms,” “generalized bullous fixed drug eruption,” “Stevens-Johnson syndrome” and “toxic epidermal necrolysis” (Online Supplement 1). Documentation and training were not systematically analyzed. Languages were limited to English and German. Pediatric populations (age below 18 years) were excluded. After May 2022, literature was added using multiple non-systematic reviews. Cross-referencing was used when appropriate, and major CM guidelines were added. The literature searches produced 328 and 480 hits, respectively. In total, 25 and 19 studies were included for this final review by two experienced authors (A.J.v.d.M., A.A.J.M.v.d.V.), based on full-text evaluation. This extensive literature review provided the base for the consecutive guideline development process following the CMSC Working Rules (www.esur.org). The concept guideline was discussed and agreed upon at a meeting of the members of the CMSC in September 2023 in Rome (Italy).

## Terminology and classification of hypersensitivity reactions to contrast media

An adverse drug reaction (ADR) after CM administration, whether iodine-based contrast media (ICM), gadolinium-based contrast agents (GBCA) or ultrasound contrast agents (USCA), is defined as a noxious and unintended response to a CM. This response suggests a reasonable possibility of a causal relationship between the CM and the adverse event.

**Fig. 1:**
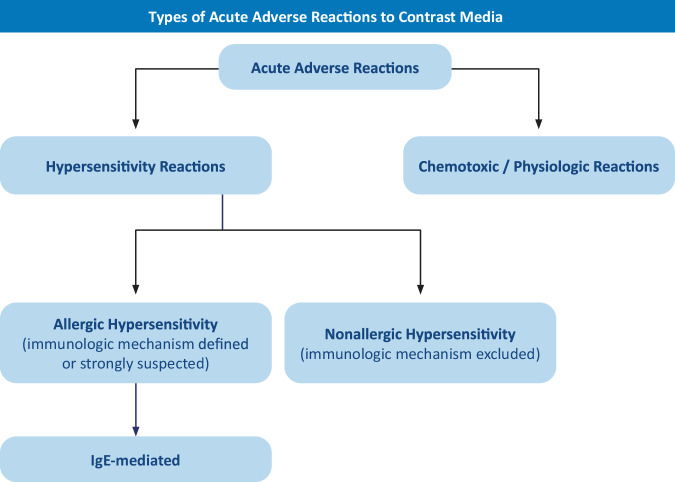
Types of acute adverse reactions to contrast media. IgE, Immunoglobulin E

ADR can be divided into Type A reactions, which are predictable and related to an exaggeration of the normal pharmacological action, and Type B or HR, which are unpredictable and not expected from the pharmacological action (Fig. 1) [1]. This CMSC guideline update will focus on HR, excluding Type A reactions. Recommendations on Type A reactions to CM are available in other CMSC documents.

HR are classified based on timing: IHR occurs within 1 h of CM administration (rarely up to 6 h), while NIHR occurs between 1 h and 1 week (rarely up to 8 weeks) after administration [2]. IHR and NIHR can be either allergic or non-allergic, see sections on pathophysiology below.

Hypersensitivity reactions to CM have been graded as mild, moderate, or severe. A practical classification of immediate hypersensitivity reactions to contrast media that is used in Radiology departments in many countries is the American College of Radiology (ACR) classification [2], previously adopted by the ESUR [3] (Table 1).

**Table 1:** Symptoms of hypersensitivity reactions and how to grade them according to the ACR [2] and Ring-Messmer classification [4]

Clinical symptoms of the hypersensitivity reaction	ACR classification	Ring and Messmer classification
Nasal congestion	Mild	Grade 1
Sneezing, conjunctivitis, rhinorrhoea		Grade 1
Limited/scattered urticaria and pruritus		Grade 1
Cutaneous oedema		Grade 1
Limited itchy/scratchy throat		Grade 1
Diffuse urticaria and pruritus	Moderate	Grade 1
Diffuse erythema, no hypotension		Grade 1
Facial oedema, no dyspnoea		Grade 1
Throat hoarseness, no dyspnoea		Grade 2
Mild bronchospasm, no hypoxia		Grade 2
Diffuse/facial oedema, with dyspnoea	Severe	Grade 2
Diffuse erythema, with hypotension		Grade 3
Laryngeal oedema, with hypoxia		Grade 3
Severe bronchospasm, with hypoxia		Grade 3
Anaphylactic shock		Grade 3
Cardiopulmonary arrest		Grade 4

Use the most intense symptom for correct classification

*ACR* American College of Radiology

In allergology, different classifications focusing on hypersensitivity reactions may be used, including the (modified) Ring-Messmer classification (Table 2) [4]. Although the ACR and Ring-Messmer classifications do not entirely overlap, with the ACR classification seeming somewhat arbitrary regarding allergic mechanisms and symptoms, here we use the ACR classification since, for radiology employees, this classification is more practical and clearly discriminates reactions that require action from those that do not.

**Table 2:** Modified Ring-Messmer classification of systemic reactions [4]

Grade	Skin	Abdomen	Airways	Cardiovascular
I	Itch Flush Urticaria Angioedema	-	-	-
II	Itch Flush Urticaria Angioedema	Nausea Cramps	Rhinorrhoea Hoarseness Dyspnoea	Tachycardia (> 20 bpm rise) Hypotension (> 20 mm Hg drop in systolic blood pressure) Arrhythmia
III	Itch Flush Urticaria Angioedema	Vomiting Defecation	Laryngeal edema Bronchospasm Cyanosis	Shock
IV	Itch Flush Urticaria Angioedema	Vomiting Defecation	Respiratory arrest	Cardiac arrest

Classification according to the most severe symptom, no symptom is mandatory

*bpm* beats per minute, *Hg* mercury

### Anaphylaxis

According to the definition agreed upon by the Global Allergy and Asthma Excellence Network (GA2LEN) in 2024, anaphylaxis is a serious hypersensitivity reaction that can progress rapidly and may lead to death. It may involve the skin/mucosa (including the lips/tongue), respiratory, cardiovascular, and/or gastrointestinal systems. Life-threatening anaphylaxis can occur without skin/mucosa involvement [5].

Anaphylaxis is a life-threatening allergic reaction, occurring rapidly after exposure to an antigen to which the patient presents some kind of intolerance. It is a multifactorial response in which multiple immunologic mechanisms are involved. The typical mechanism results from IgE-mediated activation of mast cells and basophils [6–8].

## Immediate hypersensitivity reactions (IHR) to contrast media

### Pathophysiology

HR to nonionic CM is a heterogeneous disease. These can develop from multiple mechanisms, such as IgE-dependent, complement-dependent, direct membrane effects of CM, and possibly other mechanisms that have not been identified yet [9–11].

HR may or may not be truly IgE-mediated. In general, allergy can be either antibody- or cell-mediated. Cell-mediated reactions usually occur after one or several days (see pathophysiology of NIHR), while antibody-mediated reactions tend to be more immediate. A well-known reason for immediate reactions is the presence of antigen-specific IgE antibodies attached to FcεR1 receptors on the surface of mast cells and basophils. After cross-linking of IgE antibodies on the cell surface, degranulation follows, resulting in the release of histamine and many other mediator substances. Other stimuli can also cause degranulation via other surface receptors, such as the degree of ionization, osmolality, and temperature of the injected solution. Sometimes histamine release can occur via non-IgE-dependent direct activation of the mast cell, such as via MRGPRX2 (Mas-related G-protein-coupled receptor X2)-receptors [12–14].

### Incidence and risk factors

The incidence of all HR is highest after ICM and lowest after USCA. The incidence of IHR has declined considerably after the introduction of low-osmolar and iso-osmolar ICM and the use of lower mean CM doses [2, 3].

Recent studies with large patient cohorts focusing specifically on HR after ICM have shown frequencies of 0.15 to 0.69%, with severe reactions occurring in 0.005 to 0.013% [15, 16].

HR after nonvascular CM administration (oral, rectal, intraductal, intravesical, intra-articular) are rare [17–20]. Such reactions occur more delayed, and the incidence is much lower than after intravascular administration. These will be influenced by the integrity and condition of the endothelium of the compartment into which the contrast agent is administered. Severe reactions can occur, even with nonvascular CM administration [19, 20].

Risk factors for HR to ICM can be categorized into patient-related, CM-related, and procedure-related factors. These factors are not yet fully defined and remain a topic of ongoing debate. Most available data do not clearly distinguish between ADR and HR, and are derived from non-controlled, retrospective observational studies that lack comparisons with appropriately matched control groups. Although multiple potential risk factors for HR have been described, it must be emphasized that most patients with these risk factors do not experience any reaction upon administration of CM [21].

In an overview of risk factors for HR after ICM use (Online Supplement 2), the most consistent factor predicting a future HR is a history of a previous HR to ICM (OR 27.6–198.8) [21–23], which is reflected in all guidelines [2, 3, 24–27]. A history of (drug or other) allergic diseases has lower and less consistent predictive values (OR 1.21–6.8) [21–23, 28]. Numerous minor factors such as female sex, age below 50 years, history of (uncontrolled) asthma or chronic urticaria, and multiple co-morbidities (e.g., cardiovascular, oncologic, or chronic liver disease) are less consistent and have weaker predictive values (OR 1.45–3.0) [21–23, 28] but are used additionally in specific clinical guidelines [27]. The risk from co-morbidities may be attributable to the fact that these patients often undergo multiple imaging procedures with ICM, rather than an inherent predisposition, or may be due to the use of immunomodulatory medications [28, 29]. Anyway, the risk of a history of multiple well-documented allergies or minor risk factors can be additive, leading to a more severe clinical presentation, which may be particularly relevant in unstable patients.

The risk of inducing HR by the type of nonionic ICM remains inconclusive. Whereas individual studies found moderate predictive values for (severe) IHR (OR 3.1–6.8) for iomeprol and iopromide with variable comparators [21, 22, 30], a recent meta-analysis did not have sufficient data for analysis of HR [31].

Recent studies in large adult patient cohorts focusing on HR after GBCA have shown low incidence rates of 0.06–0.17%, with severe reactions occurring in 0.003–0.006% [32–35]. In the largest Korean cohort, incidence was higher, for IHR 0.4% and for NIHR 0.04% [34].

What is outlined above for risk factors for HR to ICM also applies to GBCA. In an overview of risk factors for HR after GBCA use (Online Supplement 3), a prior HR to GBCA had the highest risk (12-fold higher frequency) [36]. A history of allergic diseases had a moderate predictive value (OR 2.83) [37]. Minor factors like female sex, age below 50 years, type of MRI, and outpatient status have weaker predictive values (OR 1.4–2.0) [36–38].

The specific GBCA agent seems more important with higher frequencies or predictive values (OR 3.0–8.0) for gadobutrol, gadoteridol, gadobenate, and gadoxetate [34, 36–38]. In a meta-analysis, the overall rate of IHR was 0.09%, with severe reactions occurring in 0.005%. Linear nonionic GBCA had a lower rate (0.015%), while nonionic macrocyclic GBCA (0.16%) and linear ionic GBCA with protein-binding (0.17%) had higher IHR rates [35].

### Management of IHR

It is important to recognize early warning signs of (a progressive) HR such as erythema, rash, or angioedema, that may be followed by a rise of respiratory frequency, increase of the heart rate, and loss of blood pressure.

Four non-comparative studies on IHR to a variety of contrast media described treatment effects in small subgroups. Most patients were discharged in good condition, but some had side effects of treatment or resolution of symptoms needing longer than 24 h [15, 39–41].

**Fig. 2:**
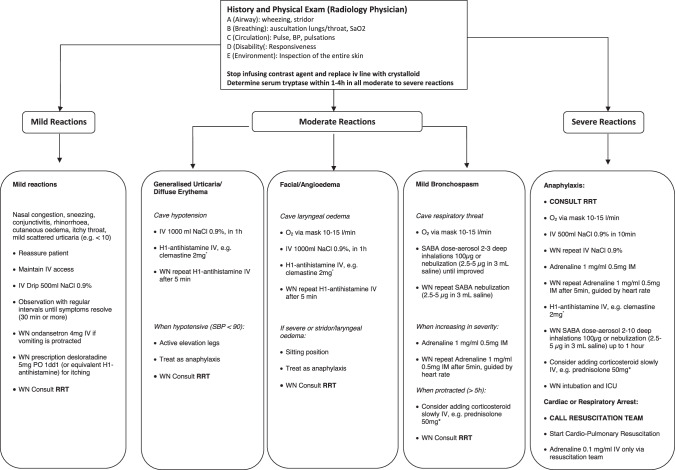
Therapy of immediate hypersensitivity reactions in adults. * Or alternative (see Table 3). Adrenaline may be less effective in case of beta-blocker use, and using glucagon or a beta-agonist such as dobutamine may be a better option. ACR, American College of Radiology; BP, blood pressure; ICU, intensive care unit; IM, intramuscular; IV, intravenous; NaCl, sodium chloride; PO, orally; RRT, rapid response team; SABA, short-acting beta-agonists; SBP, systolic blood pressure; SaO_2_, oxygen saturation; WN, when needed

Due to a lack of well-designed comparative studies, management recommendations can only be based on results of observational studies and reviews [42–48], and on the recommendations in other guidelines, such as the American College of Radiology Manual on Contrast Media v2024 [2], the European Society of Urogenital Radiology CMSC guidelines v10 [3], the World Allergy Organization (WAO) Anaphylaxis Guidelines 2020 [7] and the AAAAI/ACAAI Anaphylaxis Practice Parameter Update 2024 [8], the International Consensus On Drug Allergy 2014 [10], the guideline Safe Use of Contrast Media, Part 2, of the Radiological Society of the Netherlands [17], the European Association for Allergy and Clinical Immunology (EAACI) Anaphylaxis Guidelines 2021 [49], and the Resuscitation Council UK guidelines on anaphylaxis [50] (see Fig. 2). It is important to realize that such guidelines are regularly updated.

## Non-immediate hypersensitivity reactions (NIHR) to contrast media

### Pathophysiology

There is evidence that drug-specific T-cells play an important role in NIHR. In skin reactions, an infiltrate in the dermis consisting of activated CD4^+^ or CD8^+^ T-cells and eosinophils is usually found [51, 52]. It has been postulated that CM may not induce a primary immune response but instead interact with receptors on activated memory T-cells raised against other foreign substances (non-allergic NIHR).

In vitro studies have shown different pathways for CM recognition, both requiring major histocompatibility complex (MHC) molecules for stimulation: (1) the (pro)hapten concept, in which the CM undergoes uptake and processing by antigen-presenting cells, followed by presentation to T-cells via MHC-II molecules, and (2) the p-i concept, involving the direct binding of unprocessed CM to the T-cell receptor or MHC molecule [53]. This hapten-independent pathway would act as a pharmacological interaction of CM with immune receptors and could best explain the cross-reactivity between CM, as CM-specific activated T-cell clones would respond to shared structural elements.

### Incidence and risk factors

The incidence in published studies varies widely, but there is a lot of underreporting in daily practice and in studies. The main risk factors for NIHR to ICM are a previous HR and/or, to a lesser extent, IL-2 immunotherapy [54]. Most CM-associated non-allergic type cutaneous NIHR are associated with iso-osmolar (dimeric) CM [2, 54, 55]. Patients with NIHR are not at higher risk for an IHR (mediated by IgE or other mechanisms) upon re-exposure to CM, as these reactions are mechanistically unrelated [56].

### Management of NIHR

There are no comparative study data with a control group on different management strategies for NIHR to contrast media. In many patients, there are non-specific symptoms, such as headache, nausea, dizziness, gastrointestinal complaints, mild fever and musculoskeletal pain [51, 54, 57]. Skin rashes, skin redness, skin swelling, and headache are the most frequent symptoms of a true NIHR [58]. Rashes are generally macular or maculopapular exanthemas (MPE), which usually occur 2–10 days after first exposure to a CM and 1 to 2 days after re-exposure to the same CM. Most skin reactions are mild to moderate in severity, are usually self-limiting, and resolve within 1 week.

Discrimination should be made between mild-to-moderate reactions and the very rare SCAR with danger signs, such as acute generalized exanthematous pustulosis (AGEP), drug reaction with eosinophilia and systemic symptoms (DRESS), generalized bullous fixed drug eruptions (GBFDE), Stevens-Johnson syndrome (SJS), and toxic epidermal necrolysis (TEN) [58–60].

Treatment is symptomatic, based on the type of reaction presented. More than 90% of the NIHR involve the skin only. Usually, oral H1-antihistamines or corticosteroids, in combination with topical corticosteroid creams or foams, can be used to alleviate MPE reactions. Antipyretics may be given for fever, and anti-emetics for nausea. Any patient with (suspicion of) a SCAR should be promptly referred to a dermatologist (or drug allergy specialist) for diagnosis, treatment, and advice on CM use for future imaging.

## Drugs used in treatment

In addition to liberal use of supportive treatment by oxygen and volume expansion, the most frequently used medications in IHR in Europe are adrenaline, clemastine/chlorpheniramine, salbutamol, and, rarely, corticosteroids.

**Fig. 3:**
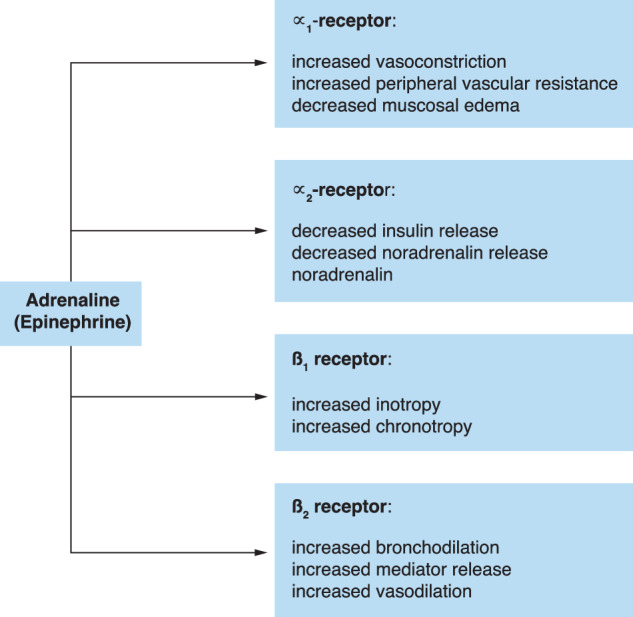
Effects of adrenaline (epinephrine)

Adrenaline (epinephrine) is a potent α- and β-adrenergic agonist with multiple systemic effects. Stimulation of α_1_-receptors leads to reduced pulmonary edema, vasoconstriction and increased peripheral vascular resistance, while β_1_-receptor activation leads to increases in inotropic and chronotropic effects on the heart and β_2_-receptor activation to bronchodilatation, vasodilatation, and reduced mediator release (Fig. 3). In low doses adrenaline activates primarily β-adrenergic receptors with mainly cardiovascular effects, while in higher doses (0.5 mg and higher) both α- and β-adrenergic receptors are activated. Even though there was no hard evidence for its use in anaphylaxis in systematic reviews of randomized trials [61], evidence from non-randomized studies is solid, and intramuscular (IM) adrenaline 1 mg/mL remains the first-line drug in severe IHR (anaphylaxis) in most guidelines, and most of these recommend an adult dose of 0.5 mg IM [7, 8, 50, 62]. Because of the diminished frequency of IHR and lack of experience in treatment, major guidelines recommend restricting adrenaline administration by non-experienced users to IM administration. Instead of adrenaline 1 mg/mL for IM administration, each department may also opt for selecting adrenaline auto-injectors [63], but most adult versions inject a lower dose of 0.3 mg. In patients that are premedicated with beta-blockers (cardiac imaging), adrenaline may not be sufficiently active and glucagon or dobutamine are better alternatives.

**Fig. 4:**
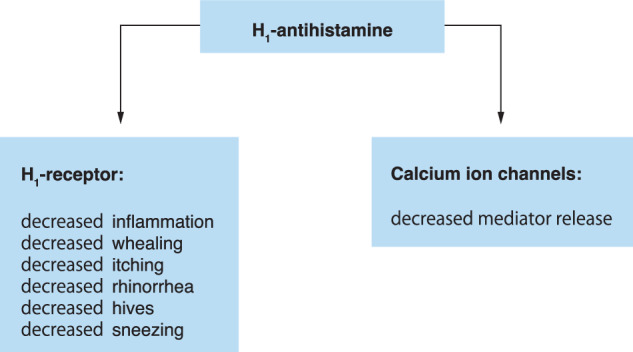
Effects of H1-antihistamines. Ca, calcium; NF-κB, nuclear factor kappa-B

Histamine H1-receptor activation can cause nasal congestion, flushing, headache, pruritus, as well as coronary and bronchial constriction. H1-antihistamines block histamine H1-receptors on various effector cells, blocking the effect selectively via competitive inhibition of the histamine H1-receptor [8]. However, mast cells and basophils secrete various other active mediators that are not blocked. H1-antihistamines can reduce mast cell mediator release, inflammation, itching, sneezing, rhinorrhoea and hives (Fig. 4). In a Cochrane review, no good evidence for its use in anaphylaxis could be found [64]. The main side effect of the old H1-antihistamines available for intravenous (IV) administration, such as clemastine or chlorpheniramine, is drowsiness/sedation. After administration, driving or equipping machines is no longer safely possible or covered by insurance. In newer nonsedating H1-antihistamines, this effect is mild, but these are only available for oral administration.

An inhaled short-acting β_2_-agonist (SABA) such as salbutamol or albuterol is a potent β_2_-receptor agonist that is rapidly distributed through the body. It has a local bronchodilator but also a systemic effect. It can effectively decrease dyspnoea and wheezing from allergic bronchospasm and reduce histamine release in the lung. The administration may be easier using nebulization to avoid stress for the patient who should inhale deeply.

Corticosteroids have various effects on the immune system, including mast cells, and therefore can block both mast cell degranulation by upregulating inhibitory signaling receptors, and inhibit cytokine production through suppression of gene transcription. In addition, they can also exert direct membrane-stabilizing effects at high concentration, which can be non-specific or specific [65]. Because of their delayed onset of action, corticosteroids play no role in the acute management of HR after CM. There is no evidence from high-quality studies for the use of corticosteroids in the emergency management of severe HR [65, 66]. Also, the beneficial effect of corticosteroids on biphasic anaphylaxis is controversial [67].

## Documentation of hypersensitivity reactions to contrast media

As the allergic evaluation by a drug allergy specialist is becoming more important in the prevention of recurrent HR, accurate agent-specific documentation is more important than ever. A model of a referral letter to the allergologist is provided in Online Supplement 4.

Documentation in the electronic health record (EHR) is far from standardized and is often done by physicians without any experience in the administration of contrast media [68–70]. Recommendations for improved documentation have recently been published [71, 72].

It is highly recommended that the physician responsible for the administration of the CM or the drug allergy specialist ensures that the allergy is accurately documented in the EHR (Table 3):

- The location, date, and time of CM administration.
- The *specific* (IV and/or oral) contrast agent’s name and dose (volume, concentration).
- The type (immediate or non-immediate) and severity classification of the HR.
- All available patient symptoms and vital signs (blood pressure, heart rate, respiration rate, oxygen saturation), time of first symptoms, and their evolution.
- The treatment given and the response of the patient to the treatment.
- Any results of a telephonic consultation with a drug allergy specialist.
- Any clinical follow-up and advice for a referral to a drug allergy specialist.

**Table 3:** Recommendations of the ESUR Contrast Media Safety Committee (based on the reaction severity classification of the ACR)

***Immediate/acute hypersensitivity reactions in adults***
What to do before the examination:
• Be prepared to treat acute adverse reactions: be knowledgeable about the risks and potential reactions, and participate regularly in continuous medical education. • Know your patient and evaluate the risk/benefit ratio and consider imaging alternatives. • If the patient has experienced a previous hypersensitivity reaction, identify the specific agent that elicited the previous reaction, and if the injection is mandatory, choose an alternative contrast agent (see further in Part 2). • Schedule the patient during daytime hours, when most personnel are present, preferably in a hospital-based CT or MRI facility. • A regularly maintained resuscitation trolley should be available in the department. • The following first-line emergency equipment and medications should be available in the examination room: Oxygen IV fluids: normal saline or Ringer lactate (stock at least 2L) Sphygmomanometer One-way mouth rebreather apparatus Adrenaline 1 mg/mL (1:1000) H1-Antihistamine suitable for injection Atropine Short-acting β2-agonist metered dose inhaler or nebulizer Anti-convulsive drugs (midazolam) • Emergency numbers for the hospital resuscitation team should be in the examination room. • Adhere to local protocols for accessibility of a resuscitation and emergency response team. • Equipment for collecting blood for tryptase measurement should be readily available (see Part 2). *Note: In patients that are premedicated with beta-blockers (cardiac imaging), adrenaline may not be sufficiently active, and glucagon or dobutamine are additional options*.
What to do during the examination:
• Monitor the patient closely and judge for progression of the reaction. • Observe closely for mucosal edema of the nose, mouth, throat, or larynx. • If there is an increased risk of an adverse reaction, a venous access should be kept in place. • In case of symptoms, check heart rate, arterial blood pressure, and consciousness. • If marked bradycardia is present, consider treatment of a vasovagal reaction (atropine). • Be aware that a patient with impaired vision or hearing, neurological disease, psychiatric illness, cognitive disorders or under CNS-active medications might have diminished awareness of hypersensitivity symptoms.
Acute management general principles:
• Check and stabilize the patient according to the ABCDE method. • Stop infusing contrast agent and replace the IV line with crystalloid. • Dyspnoea or stridor: let the patient sit up. • Hypotension: keep patient in prone position, raise legs. • Consider measuring serum tryptase, ideally 1–2 h after start of the reaction. • Record acute allergic reactions and the culprit contrast medium in the allergy registry of the electronic health record of the patient. *Note: After administration of a sedating H1-antihistamine, the patient may no longer be able (or insured) to drive a car/motorcycle or to operate machinery*.
Severe reactions (anaphylaxis):
Cardiac or respiratory arrest:
• Call the CPR team. • Start CPR.
Anaphylactic reaction or stridor:
• Call the rapid response team. • Give oxygen 10 to 15 L/min with non-rebreathing mask. • Give 0.5 mg adrenaline IM in the lateral upper thigh, repeat as necessary, guided by heart rate. • Give fluid bolus of crystalloid 500 mL IV in 10 min, repeat as necessary. • Short-acting β2-agonist: 2–10 inhalations of 100 µg of salbutamol via inhalation [depending on severity], with option to repeat every 20 min, or via nebulization (2.5–5 µg diluted in 3 mL of saline solution) up to 1 h. • Give chlorphenamine 20 mg or clemastine 2 mg IV, repeat as necessary. • Consider adding corticosteroid (for example, prednisolone 50 mg IV*). *Note: Adding corticosteroids may be beneficial to prevent protracted or biphasic anaphylactic reactions if initial symptoms are severe*.
*50 mg prednisolone IV is equivalent to:
• 40 mg methylprednisolone. • 8 mg dexamethasone. • 200 mg hydrocortisone. (Source: Brunton LL et al (eds) (2017) Goodman and Gilman’s The pharmacological basis of therapeutics, 13th edn. McGraw-Hill, New York).
Moderate reactions:
Consider transferring the patient to a department with facilities for monitoring vital functions.
Mild bronchospasm:
• Short-acting β2-agonist: 2–4 inhalations of 100 µg of salbutamol via inhalation (depending on severity), with option to repeat every 20 min, or via nebulization (2.5–5 µg diluted in 3 mL of saline solution) until clinical improvement. • In case of deterioration, give adrenaline 0.5 mg IM and consider consulting the rapid response team. • When bronchospasm increases in severity, consider repeating adrenaline 0.5 mg IM, guided by heart rate.
Facial edema without stridor:
• Give oxygen 10 to 15 L/min via a non-rebreathing mask. • Give chlorphenamine 20 mg or clemastine 2 mg IV. • If edema is severe, near airways, or if stridor develops, treat as anaphylaxis.
Diffuse urticaria/diffuse erythema:
• Give chlorphenamine 20 mg or clemastine 2 mg IV. • If accompanied by hypotension, treat as anaphylaxis.
Mild reactions:
General:
• Mild reactions may only need reassurance. • Observe vital signs until symptoms resolve. • Do not remove IV access during observation.
Consider:
• Prescribing a nonsedating antihistamine, for example, desloratadine 5 mg PO or cetirizine 10 mg PO (once daily) for mild allergic reactions. • Ondansetron 4 mg IV for protracted vomiting.
What to do after the examination:
• If the patient has experienced a mild reaction, keep him under surveillance until every symptom has disappeared (minimum 30 min, usually < 60 min). • Patient experiencing a non-life-threatening reaction should be observed for 4–6 h after successful treatment and discharged. • Patient experiencing a life-threatening reaction should be admitted and observed. • Order serum tryptase within 1–4 h (see Part 2).
***Non-immediate/delayed hypersensitivity reactions in adults***
Warn patients who have had a previous non-immediate hypersensitivity reaction to contrast media that a repeat non-immediate hypersensitivity reaction may be possible, usually a skin reaction.
Patients should contact their general practitioner if they have a non-immediate hypersensitivity reaction after contrast media administration. Consider informing the radiology department where the contrast medium was administered about the occurrence and symptoms of a non-immediate hypersensitivity reaction after contrast media administration.
When the symptoms of a non-immediate hypersensitivity reaction are mild, a wait-and-see approach can be justified.
Treat non-immediate hypersensitivity reactions symptomatically. Consider the treatment of skin reactions with oral or topical corticosteroids.
When severe cutaneous adverse reactions (SCAR) develop, such as generalized pustulosis or painful cutaneous blisters, refer the patient to a dermatologist for treatment and advice on future use of contrast media.
***Documentation of hypersensitivity reactions***
The physician responsible for the administration of the CM or the drug allergy specialist ensures that the allergy is accurately documented in the Electronic Health Record:
• The location, date, and time of CM administration • The *specific* contrast agent’s name and dose (volume, concentration) • The type (immediate or non-immediate) and severity category of the HSR • All available patient symptoms and vital signs (blood pressure, pulse, respiration rate, oxygen saturation), time of first symptoms, and their evolution • The treatment given and the response of the patient to the treatment • Any results of a telephonic consultation with a drug allergy specialist • Any clinical follow-up and advice to see a drug allergy specialist
If a HR to a contrast medium is serious or unexpected, the physician responsible for its administration or the drug allergy specialist should report all details of such a reaction to the Marketing Authorization Holder and/or the National Pharmacovigilance Authority.

*ACR* American College of Radiology, *ESUR* European Society of Urogenital Radiology, *CNS* central nervous system, *CPR* cardiopulmonary resuscitation, *HR* hypersensitivity reaction, *IM* intramuscular, *IV* intravenous, *PO* orally, *SaO*_*2*_ oxygen saturation, *SCAR* severe cutaneous adverse reaction

The radiologist should describe in detail the clinical process and how the HR evolves (see also Tables 1 and 2). It is essential that this reporting should be based on the name of the specific or culprit contrast agent (both IV and/or oral) and be done by radiologists or drug allergy specialists with experience in the use of contrast media. If a HR to a contrast medium is serious or unexpected, the physician responsible for its administration or the drug allergy specialist should report all details of such a reaction to the Marketing Authorization Holder and/or the National Pharmacovigilance Authority.

Appropriate patient information leaflets on the various radiological examinations with contrast medium administration should be available. In these leaflets, the possibility of occurrence of a NIHR should be mentioned, and detailed patient instructions should be given.

## Training of personnel

There are now fewer opportunities for physicians to recognize and appropriately treat such reactions. These reactions are often unpredictable, can vary from very mild itching to anaphylactic shock, and can happen to people who have never been exposed to contrast media. Although a mild reaction is often self-limiting, it can evolve into a severe reaction. It is important that personnel know how to judge the progression of a reaction, and in progressive reactions, call for assistance and administer adrenaline early. In many instances, adrenaline or salbutamol is underused and/or incorrectly dosed [73].

When a hypersensitivity reaction to a contrast medium occurs, there may be insufficient time or opportunity to study the treatment protocols and medication doses. It is therefore important for personnel to be prepared for any adverse reaction, to have access to clear treatment guidelines and cards/flowcharts, and to have a rapid response team in case of an emergency [74]. A regularly checked resuscitation trolley with emergency equipment and medications should always be readily available.

Therefore, personnel who work in departments where CM is administered to patients need to be regularly trained in the management of HR and other emergency situations, whereby high-fidelity hands-on simulation training programs are more effective than didactic lectures or computer-based e-learning [75–77].

## Conclusion

HR to contrast media occurs infrequently and can be either immediate or non-immediate. In imaging, most are mild or moderate, while severe reactions are very rare. The main drugs for the treatment of individual reactions include H1-antihistamines and intramuscular adrenaline, with limited indications for SABA. As these reactions occur infrequently, these situations often create stress and confusion. Therefore, detailed documentation of such reactions by personnel with experience in handling contrast media and regular hands-on training for all personnel involved in managing these reactions is mandatory. The CMSC recommendations for the management of IHR and NIHR, using the ACR classification [2], are summarized in Fig. 2 and Table 3. For prevention of recurrent HR to contrast media, see Part 2 of this guideline [78].

## Supplementary information

ELECTRONIC SUPPLEMENTARY MATERIAL

## Abbreviations

ACR: American College of Radiology
ADR: Adverse drug reaction
CM: Contrast medium/media
CMSC: ESUR Contrast Media Safety Committee
EHR: Electronic health record
ESUR: European Society of Urogenital Radiology
GBCA: Gadolinium-based contrast agent/agents
HR: Hypersensitivity reaction
ICM: Iodine-based contrast medium/media
IHR: Immediate hypersensitivity reaction
IM: Intramuscular
IV: Intravenous
MHC: Major histocompatibility complex
MPE: Maculopapular exanthema
NIHR: Non-immediate hypersensitivity reaction
SABA: Short-acting β2-agonist/agonists
SCAR: Severe cutaneous adverse reaction/reactions
USCA: Ultrasound contrast agent/agents
