# Supplementary material for: Hypersensitivity reactions to contrast media: Part 1. Management of immediate and non-immediate hypersensitivity reactions in adults. Updated guidelines by the ESUR Contrast Media Safety Committee
Source: Eur Radiol. 2025 May 27;35(11):6798–810. doi: 10.1007/s00330-025-11675-1 (PMC12559081; doi:10.1007/s00330-025-11675-1)
Supplement: Supplementary file 1 — ELECTRONIC SUPPLEMENTARY MATERIAL [file 330_2025_11675_MOESM1_ESM.pdf]

Hypersensitivity Reactions to Contrast Media: Part 1. Management of Immediate and Non-immediate Hypersensitivity Reactions in Adults. Updated ESUR Contrast Media Safety Committee Guidelines

ELECTRONIC SUPPLEMENTARY MATERIAL

Online Supplement 1: Original Literature Searches

Literature Search IHR

Database	Search String
PubMed<br><br>1985 – May 2022	("Contrast Media"[Mesh] OR contrast medi* [tiab] OR contrast agent* [tiab] OR contrast material* [tiab] OR contrast dose [tiab] OR contrast doses [tiab] OR contrast dosage [tiab] OR radiocontrast medi* [tiab] OR radiocontrast agent* [tiab] OR radiopaque medi* [tiab] OR radiocontrast dose [tiab] OR radiocontrast doses [tiab] OR radiocontrast dosage [tiab] OR "Barium"[Mesh] OR barium [tiab] OR gadolinium [tiab] OR microbubble* [tiab])<br>AND (("Drug Hypersensitivity"[Mesh] OR hypersensitiv* [tiab] OR allergic* [tiab] OR anaphylaxis [tiab] OR anaphylact* [tiab] OR adverse reaction*[tiab] OR urticaria* [tiab] OR diffuse erythema [tiab] OR facial edema [tiab] OR angioedema [tiab] OR bronchospasm* [tiab] OR laryngeal edema [tiab] OR anaphylactic shock [tiab] OR hypotension [tiab] OR pulmonary edema [tiab] OR cardiac arrest [tiab] OR respiratory arrest [tiab]) AND (acute [tiab] OR after administration [tiab] OR rapid* [tiab] OR severe [tiab]))<br>AND (treatment [tiab] OR treat [tiab] OR recommend* [tiab])<br>AND ("english"[Language]) AND ("1985"[Date - Publication] : "3000"[Date - Publication])
Embase (Elsevier)	contrast medium'/exp/mj OR (((contrast OR radiocontrast) NEAR/2 (medi* OR agent* OR material* OR dose OR doses OR dosage)):ab,ti) OR 'radiopaque medi*':ab,ti OR 'barium'/exp/mj OR barium:ab,ti OR 'gadolinium'/exp/mj OR gadolinium:ab,ti OR 'microbubble'/exp/mj OR microbubble*:ab,ti)<br>AND (('hypersensitivity'/exp OR hypersensitiv*:ab,ti OR allergic*:ab,ti OR anaphylaxis:ab,ti OR anaphylactic:ab,ti OR 'adverse reaction*':ab,ti OR urticaria*:ab,ti OR 'diffuse erythema':ab,ti OR 'facial edema':ab,ti OR angioedema:ab,ti OR bronchospasm:ab,ti OR 'laryngeal edema':ab,ti OR 'anaphylactic shock':ab,ti OR hypotension:ab,ti OR 'pulmonary edema':ab,ti OR 'cardiac arrest':ab,ti OR 'respiratory arrest':ab,ti) AND (acute:ab,ti OR 'after administration':ab,ti OR rapid*:ab,ti OR severe:ab,ti))<br>AND (treatment:ab,ti OR treat:ab,ti OR recommend*:ab,ti))<br>AND [english]/lim AND [1985-2018]/py<br>NOT 'conference abstract':it NOT (('animal experiment'/exp OR 'animal model'/exp OR 'nonhuman'/exp) NOT 'human'/exp)

Literature Seach NIHR

Database	Search string
PubMed<br><br>1985 – May 2022	(((((("Contrast Media"[Majr] OR contrast medi* [ti] OR contrast agent* [ti] OR contrast material* [ti] OR contrast dose [ti] OR contrast doses [ti] OR contrast dosage [ti] OR radiocontrast medi* [ti] OR radiocontrast agent* [ti] OR radiopaque medi* [ti] OR radiocontrast dose [ti] OR radiocontrast doses [ti] OR radiocontrast dosage [ti] OR "Barium"[Mesh] OR barium [tiab] OR gadolinium [tiab] OR microbubble* [tiab])))<br>AND ((("Drug Hypersensitivity"[Mesh] OR hypersensitiv* [tiab] OR allerg* [tiab] OR anaphylax* [tiab] OR anaphylact* [tiab] OR "Exanthema"[Mesh] OR exanthem* [tiab] OR rash [tiab] OR adverse reaction*[tiab] OR urticaria* [tiab] OR erythem* [tiab] OR hypotension [tiab] OR hypertension [tiab] OR "Stevens-Johnson Syndrome"[Mesh] OR stevens johnson syndrome [tiab] OR sjs [tiab] OR toxic epidermal necrolys* [tiab] OR "Drug Hypersensitivity Syndrome"[Mesh] OR dress syndrome [tiab] OR iodide mump* [tiab]) AND (late [tiab] OR delayed [tiab] OR nonimmediate [tiab])) OR late reaction* [tiab] OR delayed reaction* [tiab] OR nonimmediate reaction* [tiab])))<br>AND (("english"[Language]) AND ("1985"[Date - Publication] : "3000"[Date - Publication])))
Embase (Elsevier)	(('contrast medium'/exp/mj OR (((contrast OR radiocontrast) NEAR/2 (medi* OR agent* OR material* OR dose OR doses OR dosage)):ti) OR 'radiopaque medi*':ab,ti OR 'barium'/exp/mj OR barium:ab,ti OR 'gadolinium'/exp/mj OR gadolinium:ab,ti OR 'microbubble'/exp/mj OR microbubble*:ab,ti)<br><br>AND (('hypersensitivity'/exp OR hypersensitiv*:ab,ti OR anaphylax*:ab,ti OR allerg*:ab,ti OR 'rash'/exp OR rash:ab,ti OR 'adverse reaction*':ab,ti OR hypotension:ab,ti OR hypertension:ab,ti OR urticaria*:ab,ti OR erythem*:ab,ti OR exanthem*:ab,ti OR 'stevens johnson syndrome'/exp OR 'stevens johnson syndrome':ab,ti OR sjs:ab,ti OR 'toxic epidermal necrolysis'/exp OR 'toxic epidermal necrolys*':ab,ti OR 'dress syndrome'/exp OR 'dress syndrome':ab,ti OR 'iodide mump*':ab,ti) AND (late:ab,ti OR delayed:ab,ti OR nonimmediate:ab,ti) OR (((late OR delayed OR nonimmediate) NEAR/2 reaction*):ab,ti)))<br><br>AND [english]/lim AND [1985-2022]/py<br>NOT 'conference abstract':it NOT (('animal experiment'/exp OR 'animal model'/exp OR 'nonhuman'/exp) NOT 'human'/exp)

Online Supplement 2
Relevant studies with risk factor analyses for hypersensitivity and adverse drug reactions to iodine-based contrast media (sorted by year)

Author, Year Type of Reactions	***Origin***	***Route of CM Administration***	***Cases***	***Controls***	***Risk Factor***	***Frequency (%) / Odds Ratio (adj)***
**Hypersensitivity Reactions**						
Fukushima, 2023 *Severe IHR*	R	IV (CT)	45 (0,16%) *4 premedication*	No controls	Iomeprol vs Iopamidol	OR 6,8
McDonald, 2023 *All HR*	R	IV (CT)	1,150 (0,32%)	No controls	Age < 50 years (highest 21-30 years)	OR 1,68-2,26
					Female sex	OR 1,49
					Non-white race	OR 1,77
					Prior HR to ICM	OR 27,6
					Prior HR to GBCA	OR 1,91
					Asthma (only moderate-severe HR)	OR 1,45
					History of other allergies	OR 1,21
					Iopromide vs Iohexol	OR 3,07
					Increasing ICM dose	OR 1,30
Voltolini, 2022 *All HR*	A	Data not included	407 *400 skin tests* *78 premedication*	152	First ICM exposure	OR 2,84
					Cardiovascular diseases	OR 2,06
					History of respiratory allergy	OR 2,30
					History of adverse drug reaction	OR 1,99
Cha, 2019 *All HR*	R	Data not included	1,433 (0,73%) *541 premedication*	1,433	Iomeprol	0,95%
					Iobitridol	0,89%
					Prior HR to ICM	OR 198,8
					Hyperthyroidism	OR 3,6
					History of drug allergy	OR 3,5
					History of other allergic diseases	OR 6,8

					Family history of HR to ICM	OR 14,0
Lee, 2019<br>*Immediate HR*	A	IV<br>(CT)	2004 (0,97%)	No controls	Prior HR to ICM	OR 40,69
					Age below 50 years	OR 2,11
					Presence of asthma	OR 1,47
					Female sex	OR 1,29
					Comorbid allergic disease	2,6%
					Patients with chronic liver disease	3,1%
					Patients with cancer	2,1%
**Adverse Drug Reactions**						
Zeng, 2024<br>*All ADR*	R	IV<br>(CT)	522 (0,11%)<br>*Acute 469 (0,099%)*<br>*Delayed 53 (0,011%)*	522	Season: summer	OR 1,579
					Season: autumn	OR 1,925
					Female sex	0,141%
					Age 21-30 years	0,241%
					Iopromide 370 mgI/ml (acute ADR)	0,218%
					Iodixanol 320 mgI/ml (delayed ADR)	0,039%
Chatani, 2023<br>*All ADR*<br>*(First visit only)*	R	Data not included	163 (0,72%)	No controls	History of asthma	OR 17,4
					History of drug allergy	OR 2,4
					Outpatients	OR 2,1
					Use of premedication	OR 3,7
Kang, 2022<br>*Delayed ADR*	R	IV<br>(CT)	207 (2,0%)	7,260	Female sex	OR 1,51
					History of drug allergy	OR 4,59
					History of allergy	OR 2,54
Li, 2017<br>*All ADR*	R	IV<br>(CT)	506 (0,42%)	No controls	Female sex	0,46%
					Prior HR to ICM	7,17%
					Age 20-29 years	0.74%
					ICM dose > 100ml	0,60%
					ICM injection speed > 5ml/s	0,57%
					BMI > 24	0,47%
					IOCM use	0,69%

					Patients with asthma	2,04%
					Patients with heart failure	1,10%
					Patients with gout	0,70%
Kobayashi, 2013 *All ADR*	R	IV (CT)	Derivation: 409 (2,0%)	No controls	Prior HR to CM	OR 7,1
					Urticaria	OR 2,7
					Allergy to other drugs	OR 1,9
					ICM concentration >70%	OR 1,9
					Age < 50 years	OR 1,8
					CM Iodine dose >65g	OR 1,4

Abbreviations:
A = Allergology; ADR = adverse drug reactions; BMI = body mass index; CM = Contrast Media; CT = computed tomography; HR = hypersensitivity reactions; GBCA = gadolinium-based contrast agent; ICM = iodine-based contrast medium; IOCM = iso-osmolar contrast medium; IV = intravenous; OR = Odds Ratio; R = Radiology

References

Hypersensitivity Reactions

Fukushima Y, Taketomi-Takahashi A, Suto T, Hirasawa H, Tsushima Y (2023) Clinical features and risk factors of iodinated contrast media (ICM)-induced anaphylaxis. Eur J Radiol 164:110880.

McDonald JS, Larson NB, Schmitz JJ, et al (2023) Acute adverse events after iodinated contrast agent administration of 359,977 injections: a single-center retrospective study. Mayo Clin Proc 98: 1820-1830. DOI: 10.1016/j.mayocp.2023.02.032.

Voltolini S, Cofini V, Murzilli F, et al (2022) Hypersensitivity reactions to iodinate contrast media in Italy: a retrospective study. Characteristics of patients and risk factors. Eur Ann Allergy Clin Immunol 54: 60-67. DOI: 10.23822/EurAnnACI.1764-1489.225.

Cha MJ, Kang DY, Lee W, et al (2019) Hypersensitivity reactions to iodinated contrast media: a multicenter study of 196,081 patients.
Radiology 293: 117-124. DOI: 10.1148/radiol.2019190485.

Lee SY, Kang DY, Kim JY, et al (2019) Incidence and risk factors of immediate hypersensitivity reactions associated with low-osmolar iodinated contrast media: a longitudinal study based on a real-time monitoring system. J Investig Allergol Clin Immunol 29: 444-450. DOI 10.18176/jiaci.0374.

Adverse Drug Reactions

Zeng W, Tang J, Xu X, et al (2024) Safety of non-ionic contrast media in CT examinations for out-patients: retrospective multicenter analysis of 473,482 patients. Eur Radiol 34: 5570-5577. DOI: 10.1007/s00330-024-10654-2.

Chatani R, Kondo S, Kamimura T, et al (2023) Exploring factors affecting the occurrence of hypersensitivity reactions induced by nonionic iodine contrast media. J Clin Pharmacol 63: 1002-1008. DOI: 10.1002/jcph.2256.

Kang DY, Lee SY, Ahn YH, et al (2022) Incidence and risk factors of late adverse reactions to low-osmolar contrast media: A prospective observational study of 10,540 exposures. Eur J Radiol 146: 110101. DOI: 10.1016/j.ejrad.2021.110101.

Li X, Liu H, Zhao L, et al (2017) Clinical observation of adverse drug reactions to non-ionic iodinated contrast media in population with underlying diseases and risk factors. Br J Radiol 90(1070): 20160729. DOI: 10.1259/bjr.20160729.

Kobayashi D, Takahashi O, Ueda T, Deshpande GA, Arioka H, Fukui T (2013) Risk factors for adverse reactions from contrast agents for computed tomography. BMC Med Inform Decis Mak 13: 18. DOI: 10.1186/1472-6947-13-18.

Online Supplement 3
Relevant studies with risk factor analyses for hypersensitivity and adverse drug reactions to gadolinium-based contrast agents (sorted by year)

Author, Year Type of Reactions	***Origin***	***Route of CM Administration***	***Cases***	***Controls***	***Risk Factor***	***Frequency (%) / Odds Ratio (adj)***
**Hypersensitivity Reactions**						
Fukushima, 2024<br>*Immediate HR*	R	IV<br>(MRI)	First 67 (0,16%)<br>Repeat 8 (1,9%)<br>240 of previous HR with premedication	No controls	Gadoxetate	OR 8,03
					Gadoteridol	OR 4,93
					Gadopentetate	OR 3,27
					Younger age	OR 0,99
					Previous HR to GBCA	1,9%
					Gadobutrol (repeat HR)	6,4%
Ahn, 2022<br>*All HR*	R	IV<br>(MRI)	1,304 (0,4%)	No controls	Prior HR to ICM	OR 4,6
					Gadoteridol	0,8%
McDonald, 2019<br>*All HR*	R	IV<br>(MRI)	442 (0,16%)	No controls	Gadobenate	OR 3,9
					Gadobutrol	OR 2,3
					Female sex	OR 1,7
					Age 21-50 years	OR 1,4-1,6
					Outpatients	OR 1,9
					Abdomen-Pelvis or Prostate MRI	OR 1,4
					Chest or Cardiac MRI	OR 1,5
Jung, 2012<br>*Immediate HR*	R	IV<br>(MRI)	112 (0,08%)	No controls	Gadobenate	OR 3,00
					Female sex	OR 1,69
					History of allergies/asthma	OR 2,83
					Recurrence after prior HR	30%

**Adverse Drug Reactions**						
Granata, 2016<br>*Immediate ADR*	R	IV<br>(MRI)	32 (0,30%)<br>1785 with premedication	No controls	Gadobenate	0,50%
Aran, 2015<br>*All ADR*	R	IV<br>(MRI)	204 (0,1%)	No controls	Gadofosveset meglumine	0,8%
					Gadoxetate disodium	0,31%
					Gadobenate	0,22%
					Female sex	0,13%
					Outpatients	0,17%
					Abdomen-Pelvis or Liver MRI	0,17%
Bruder, 2015<br>*Acute ADR*	R	IV<br>(MRI)	45 (0,12%)	No controls	Gadobenate	0,42%
					MRI for viability in CAD	0,22%
Nelson, 1995<br>*All ADR*	R	IV<br>(MRI)	372 (2,4%)	No controls	History of allergies/asthma	3,7%
					Previous HR to GBCA	21,3%
					Previous HR to ICM	6,3%

Abbreviations:
ADR = adverse drug reactions; CAD = coronary artery disease; GBCA = gadolinium-based contrast agent; HR = hypersensitivity reactions; ICM = iodine-based contrast medium; IV = intravenous; MRI = magnetic resonance imaging; OR = Odds Ratio; R = Radiology

References

Fukushima Y, Ozaki D, Taketomi-Takahashi A, et al (2024) Assessment of first-time and repeated acute adverse reactions to gadolinium-based contrast agents in MRI: A retrospective study. Eur J Radiol 176: 111504. DOI: 10.1016/j.ejrad.2024.111504.

Ahn YH, Kang DY, Park SB, et al (2022) Allergic-like hypersensitivity reactions to gadolinium-based contrast agents: An 8-year cohort study of 154,539 patients. Radiology 303: 329-336. DOI: 10.1148/radiol.210545.

McDonald JS, Hunt CH, Kolbe AB, et al (2019) Acute adverse events following gadolinium-based contrast agent administration: A single-center retrospective study of 281,945 injections. Radiology 292: 620-627. DOI: 10.1148/radiol.2019182834.

Jung JW, Kang HR, Kim MH, et al (2012) Immediate hypersensitivity reaction to gadolinium-based MR contrast media. Radiology 264: 414-422. DOI: 10.1148/radiol.12112025.

Granata V, Cascella M, Fusco R, et al (2016) Immediate adverse reactions to gadolinium-based MR contrast media: A retrospective analysis on 10,608 examinations. Biomed Res Int 2016: 3918292. DOI: 10.1155/2016/3918292.

Aran S, Shaqdan KW, Abujudeh HH (2015) Adverse allergic reactions to linear ionic gadolinium-based contrast agents: Experience with 194,400 injections. Clin Radiol 70: 466-475. DOI: 10.1016/j.crad.2014.12.011.

Bruder O, Schneider S, Pilz G, et al (2015) 2015 Update on acute adverse reactions to gadolinium-based contrast agents in cardiovascular MR. Large multi-national and multi-ethnical population experience with 37788 patients from the EuroCMR Registry. J Cardiovasc Magn Reson 17: 58. DOI: 10.1186/s12968-015-0168-3.

Nelson KL, Gifford LM, Lauber-Huber C, Gross CA, Lasser TA (1995) Clinical safety of gadopentetate dimeglumine. Radiology 196: 439-443.

Online Supplement 4:

EXAMPLE OF A LETTER FOR THE PATIENT TO TAKE TO THE ALLERGY CONSULTATION

Dear Colleague,

(Insert patient's name and details) had a hypersensitivity reaction after the administration of a contrast agent on (insert location and date).

Examination type (e.g., Angiography, CT, MRI, US, Fluoroscopy):

Type of contrast agent:

- Iodine-based
- Gadolinium-based
- Ultrasound

Name of the specific contrast agent:

Concentration:

Volume administered: (xxx) ml

Route of administration (e.g., IV, IA, intra-articular, oral, rectal, local......):

Time between the injection and the start of the clinical symptoms:

Type of symptoms and their evolution (describe):

Modified Ring-Messmer classification of systemic reactions

Grade	Skin	Abdomen	Airways	Cardiovascular
I	Itch<br>Flush<br>Urticaria<br>Angioedema	-	-	-
II	Itch<br>Flush<br>Urticaria<br>Angioedema	Nausea<br>Cramps	Rhinorrhoea<br>Hoarseness<br>Dyspnoea	Tachycardia (>20 bpm rise)<br>Hypotension (>20 mm Hg drop in systolic blood pressure)<br>Arrhythmia
III	Itch<br>Flush<br>Urticaria<br>Angioedema	Vomiting<br>Defecation	Laryngeal oedema<br>Bronchospasm<br>Cyanosis	Shock
IV	Itch<br>Flush<br>Urticaria<br>Angioedema	Vomiting<br>Defecation	Respiratory arrest	Cardiac arrest
Classification according to the most severe symptom, no symptom is mandatory				

Treatment given during the reaction: • (please specify)

Outcome (e.g., follow up, return home, hospital admission, ICU,......):

Tryptase performed at the time of the reaction: Yes/No
Tryptase performed 2h after start of the reaction: Yes/No
Results: ug/ml

Previous history of contrast agent reaction: Yes/No

If yes, please specify location, date, name and type of contrast agent and symptoms:

Thank you for seeing the patient and performing an allergic study to categorize the reaction as either allergic or non-allergic hypersensitivity, and to look for cross-reactivity so that a safer contrast agent can be recommended for future injections.

Yours sincerely,

Dr (Name and details)
